# Vitamin D Deficiency in India: Prevalence, Causalities and Interventions

**DOI:** 10.3390/nu6020729

**Published:** 2014-02-21

**Authors:** Ritu G, Ajay Gupta

**Affiliations:** 1 Charak Foundation, P.O. Box 3547, Cerritos, CA 90703, USA; 2 Rockwell Medical Inc., 30142 S. Wixom Road, Wixom, MI 48393, USA

**Keywords:** vitamin D, vitamin D deficiency, 25-hydroxyvitamin D, India, Indian subcontinent, healthy individuals, fortification strategies, supplementation, sun exposure, osteoporosis, fractures

## Abstract

Vitamin D deficiency prevails in epidemic proportions all over the Indian subcontinent, with a prevalence of 70%–100% in the general population. In India, widely consumed food items such as dairy products are rarely fortified with vitamin D. Indian socioreligious and cultural practices do not facilitate adequate sun exposure, thereby negating potential benefits of plentiful sunshine. Consequently, subclinical vitamin D deficiency is highly prevalent in both urban and rural settings, and across all socioeconomic and geographic strata. Vitamin D deficiency is likely to play an important role in the very high prevalence of rickets, osteoporosis, cardiovascular diseases, diabetes, cancer and infections such as tuberculosis in India. Fortification of staple foods with vitamin D is the most viable population based strategy to achieve vitamin D sufficiency. Unfortunately, even in advanced countries like USA and Canada, food fortification strategies with vitamin D have been only partially effective and have largely failed to attain vitamin D sufficiency. This article reviews the status of vitamin D nutrition in the Indian subcontinent and also the underlying causes for this epidemic. Implementation of population based educational and interventional strategies to combat this scourge require recognition of vitamin D deficiency as a public health problem by the governing bodies so that healthcare funds can be allocated appropriately.

## 1. Introduction

Vitamin D deficiency is pandemic, yet it is the most under-diagnosed and under-treated nutritional deficiency in the world [1,2,3]. Vitamin D deficiency is widespread in individuals irrespective of their age, gender, race and geography. Vitamin D is photosynthesized in the skin on exposure to UVB rays. Sun exposure alone ought to suffice for vitamin D sufficiency. However, vitamin D deficiency is widely prevalent despite plentiful sunshine even in tropical countries like India.

Vitamin D deficiency has a bearing not only on skeletal but also on extraskeletal diseases. Owing to its multifarious implications on health, the epidemic of vitamin D deficiency in India is likely to significantly contribute to the enormous burden on the healthcare system of India. Cultural and social taboos often dictate lifestyle patterns such as clothing—that may limit sun exposure and vegetarianism—which certainly limits vitamin D rich dietary options. Most Indians are vegetarians. The socioeconomically backward people constitute a large percentage of the population in India. The underprivileged generally suffer from overall poor nutrition. Vitamin D rich dietary sources are limited and unaffordable to most Indians. Vitamin D supplements are available, but most Indians are not aware that they need additional vitamin D. Additionally, the cost of these supplements is essentially prohibitive to the majority. Fortification of staple foods with vitamin D may prove to be a more viable solution towards attaining vitamin D sufficiency in India.

There are scores of research papers in the literature reporting poor vitamin D status from all over India and some from other countries of the Indian subcontinent too. These research papers have been included in this review. Many of these studies measured serum 25-hydroxyvitamin D levels in ostensibly healthy subjects. Biochemical evidences of suboptimal bone health are elevated alkaline phosphatase (ALP) and elevated PTH levels (secondary hyperparathyroidism or SHPT). These were often reported in research papers, especially with respect to their correlation with vitamin D status. However, the most accurate and convincing measure of bone mineral density (BMD) is DEXA (Dual Energy X-ray Absorptiometry). Research articles reporting BMD, as measured by DEXA, in the context of vitamin D status of ostensibly healthy individuals, are few. BMD data, as they correlated with the vitamin D status in ostensibly healthy Indians is compiled. A few articles also reported results of interventions studies such as vitamin D supplementation and vitamin D fortified foods.

The aim of the paper is to impress upon the practicing physicians in India about the gravity of the vitamin D deficiency problem throughout India, so that they may take necessary caution and care in the diagnosis and treatment of vitamin D deficiency. Additionally, this article may also serve to make the case to the Health ministry, and the Food and Nutrition Board in India for population based strategy of fortification of staple foods with vitamin D. This paper provides a comprehensive picture of the vitamin D status of ostensibly healthy Indians, countrywide. The most reliable marker of vitamin D status is the serum concentration of 25(OH)D. In the publications, investigators reported their data on 25(OH)D levels either as nM (nanomoles per liter) or ng/mL. To simplify information and for the ease of comparison, in this review all the data on 25(OH)D levels are presented in a single concentration unit for serum 25(OH)D levels—ng/mL. Most investigators have used different cut-off levels to define vitamin D deficiency, insufficiency and sufficiency levels. While some may have done so due to preference perhaps, other investigators defined their own cut-off levels as determined by the linear regression between 25(OH)D levels and PTH levels. Therefore, to further facilitate comparison, in this review vitamin D deficiency is defined as 25(OH)D < 20 ng/mL, insufficiency as 20–29 ng/mL and sufficiency as ≥30 ng/mL. The data compiled here are also stratified by geographic locale of the study subjects, to press home the fact that vitamin D deficiency is widespread throughout the nation, irrespective of diverse dietary and social practices. For instance, despite fish being a staple diet in Bengal (eastern India) vitamin D nutrition is no better than in other regions. Geographical stratification of data also indicates regions of the country from where more data is needed. The effectiveness or failure of vitamin D supplementation and food fortification in restoring vitamin D levels and bone health have been reviewed, while making the case that vitamin D deficiency needs to be recognized as a public health problem. This review also discusses the need and feasibility of fortification of staple foods with vitamin D in India. Steps to be taken by the policymakers in India are also mentioned. Data, when available, from other countries of the Indian subcontinent are also compiled and presented for comparison.

## 2. Vitamin D Metabolism

Vitamin D can be synthesized in sufficient amounts by most vertebrates on adequate exposure of the skin to sunlight (UVB rays). It is critical that most vertebrates obtain a sufficient amount of vitamin D either from their diet or from adequate exposure of the skin to sunlight. The term “vitamin D” refers to compounds vitamin D_3_ (cholecalciferol) or vitamin D_2_ (ergocalciferol). Vitamin D_3_ is produced in the skin on exposure to sunlight. Vitamin D_3_ is derived from 7-dehydrocholesterol by ultraviolet irradiation of the skin. Vitamin D_3_ is also found in animal food sources e.g., fatty fish (e.g., salmon, mackerel and tuna) cod liver oil, milk, *etc*. Vitamin D_2_ is found in vegetal sources like sun-exposed yeast and mushrooms. Notably, most dietary sources are not sufficiently rich in their vitamin D content.

Vitamin D (both forms D_3_ or D_2_) is a prohormone which requires two hydroxylations to finally attain its biologically active form—1,25(OH)_2_D. The first hydroxylation occurs in the liver, at position C25 to form 25-hydroxyvitamin D, also known as 25(OH)D or calcidiol. 25(OH)D is the major circulating form of vitamin D. The second hydroxylation occurs at position C1α to form 1,25(OH)_2_D, also known as calcitriol. 1,25(OH)_2_D is produced primarily but not exclusively in the kidneys. 1,25(OH)_2_D is released in blood, where it binds to vitamin D binding protein (DBP) and reaches its target tissues to exert its endocrine functions through the vitamin D receptor (VDR). 1,25(OH)_2_D is also produced in several extrarenal tissues for its paracrine and autocrine functions. Most cells in the body have VDR. Many cell types can also produce 1,25(OH)_2_D. 1,25(OH)_2_D is capable of regulating a wide variety of genes that have important functions in regulating cell growth and differentiation.

## 3. Vitamin D and Skeletal Health

Rickets, osteomalacia and osteoporosis are widely prevalent all over the world. The most well recognized function of 1,25(OH)_2_D involves regulation of calcium and phosphorus balance for bone mineralization and remodeling. Without adequate levels of 1,25(OH)_2_D in the bloodstream, dietary calcium cannot be absorbed. Low calcium levels lead to an increase in serum PTH concentration, which leads to increased tubular reclamation of calcium in kidneys and resorption from the skeleton at the cost of lowering bone density. In the long term this leads to weakened and brittle bones that break easily. Approximately 40%–60% of total skeletal mass at maturity is accumulated during childhood and adolescence. Rickets results from inadequate mineralization of growing bone. Thus it is a childhood disease and it is manifested as bone deformities, bone pain and weakness. Biochemical abnormalities consistently include hypophosphatemia, elevated alkaline phophatase levels and serum 25(OH)D levels are usually below 5 ng/mL. Chronic vitamin D deficiency in adults results in osteomalacia, osteoporosis, muscle weakness and increased risk of falls [4,5,6,7,8,9,10,11]. Epidemiological support for skeketal benefits of vitamin D is well known [5,6,12,13,14].

## 4. Vitamin D: Extraskeletal Effects

Biochemical studies have implicated vitamin D deficiency in many chronic diseases including, but not limited to, infectious diseases, autoimmune diseases, cardiovascular diseases, diabetes and cancer. Numerous epidemiological publications support the extraskeletal benefits of vitamin D and they cannot be ignored even though majority of these are association studies or small randomized controlled trials. Nevertheless, stronger evidence is required with the aid of more robust and reliable statistical methods such as randomized controlled trials (RCTs). These RCTs should be well-designed, well-executed and conducted worldwide to generate dependable and incontrovertible data, in order to assess the benefits of vitamin D supplementation not only as a preventive measure but also as adjuvant therapies [12,15,16].

### 4.1. Immunity

As early as in the 19th century, cod liver oil (a rich source of vitamin D) was used for treating tuberculosis (TB). Skin exposure to sunlight was an effective therapy for treating Mycobacterium infections of the skin. In 1903, Finsen received the Nobel Prize for demonstrating that *Lupus vulgaris*, the epidermal form of TB, could be cured using light from an electric arc lamp. In early 1900s, growing awareness of benefits of sun exposure pertaining treatment of infectious diseases led to the development of sanatoriums in “sun-rich areas”. These sanatoriums enabled regimented sun exposure, diet and exercise. These sanatoriums primarily hosted TB patients [17]. Recent studies have linked vitamin D deficiency with increased risk of developing TB [18,19], otitis media [20], upper respiratory tract infections [21] and influenza [22].

### 4.2. Cardiovascular Health

Cardiovascular diseases (CVDs), including heart failure and coronary artery disease are a major cause of morbidity and mortality worldwide. There is accumulating epidemiological evidence from observational studies suggesting that CVDs are associated with vitamin D deficiency [23,24]. Increased risk of hypertension was associated with living at higher latitudes [25]. 25(OH)D level < 21 ng/mL was associated with increased risk of hypertension, diabetes, obesity and high triglyceride levels—all associated with increased cardiovascular mortality [26]. Various studies have reported reduced 25(OH)D concentrations in patients with previous and prevalent cardiovascular or cerebrovascular diseases [15,27].

### 4.3. Type 1 Diabetes

Type 1 diabetes (T1D) is caused by autoimmune destruction of pancreatic β cells, which eventually leads to insulin-dependent diabetes. Higher rates of incidence of T1D were observed at higher latitudes worldwide [28,29]. Epidemiological association of vitamin D intake and reduced risk of T1D was also seen [30]. A meta-analysis of observational studies showed a 30% reduction in risk of T1D in children receiving vitamin D supplements [31].

### 4.4. Type 2 Diabetes

Type 2 diabetes (T2D) is marked by insulin resistance (IR). In IR insulin is adequately or overproduced by pancreatic β cells, but is ineffectively utilized by the target cells of adipose, hepatic and skeletal muscles tissues. As a response to hyperglycemia, β cells further increase insulin production leading to hyperinsulinemia, which is often indicative of a pre-(T2D) stage. Hyperinsulinemia is associated with hypertension, obesity, dyslipidemia, and glucose intolerance [32]. These conditions are collectively known as “metabolic syndrome” [33]. A meta-analysis of observational studies showed inverse relation of 25(OH)D levels and calcium status with insulin resistance and hyperglycemia. In this meta-analysis, supplementation with both the nutrients combined showed benefit in optimizing glucose levels [34].

### 4.5. Cancer

Adults living at higher latitudes are more likely to develop and die of colorectal cancer [35,36], prostate cancer [37], ovarian cancer [38], breast cancer [39], lung cancer [40] and esophageal cancer [41]. Retrospective and prospective epidemiologic studies showed that when 25(OH)D levels were <20 ng/mL there was a 30%–50% increased risk of developing and dying of colorectal, prostate, breast, pancreatic, and esophageal cancer [36,39,42,43,44,45].

## 5. Serum 25(OH)D Levels as Indicative of Vitamin D Deficiency, Insufficiency or Sufficiency

Maintenance of adequate levels of serum 25(OH)D is essential to sustain the claimed pleiotropic effects, whether skeletal (classical) or extra-skeletal (non-classical). The threshold levels of serum 25(OH)D required to optimize its effects may not be the same in the various target organs. Based on classical skeletal effects, vitamin D deficiency is defined as serum levels of 25(OH)D < 20 ng/mL (50 nmol/L) with consequent and consistent elevation of PTH and reduction in intestinal calcium absorption. Vitamin D insufficiency is defined as serum 25(OH)D levels in the range of 20–29 ng/mL. At serum 25(OH)D levels of 30 ng/mL intestinal calcium absorption reaches its peak, and PTH levels continue to fall until this level of 25(OH)D is attained. Thus, vitamin D sufficiency is defined as serum levels of 25(OH)D 30–32 ng/mL. A desirable and safe range of serum 25(OH)D levels would be 30–100 ng/mL. This range would be sufficient for most known effects of vitamin D and also significantly lower to obviate concerns pertaining vitamin D toxicity.

## 6. Vitamin D Supplements and Efficacy Concerns

Commercially, vitamin D_2_ is manufactured by ultraviolet irradiation of ergosterol from yeast. Vitamin D_3_ is produced by the ultraviolet irradiation of 7-dehydrocholesterol from lanolin. Both forms of vitamin D are available as vitamin D supplements. Several other forms of vitamin D supplements are also available. 1,25(OH)_2_D is indicated specifically for patients with renal diseases and 25(OH)D is useful when hepatic hydroxylation of vitamin D is impaired.

Whether D_2_ or D_3_, is more efficacious in raising and sustaining serum 25(OH)D levels, remains controversial. Several studies have proved equivalence between the two forms [46,47,48,49]. However, other studies reported that D_3_ is more effective than D_2_ in achieving and maintaining higher serum 25(OH)D levels [50,51,52,53]. Nevertheless, on a long term basis either form may be used, bearing in mind the long half-life (2–3 weeks) of 25(OH)D in circulation.

## 7. Testing Vitamin D Status

Plasma 25(OH)D or calcidiol (a summation of D_3_ and D_2_ forms) is the most reliable marker of vitamin D status. Immunoassays such as radioimmunoassay (RIA), enzyme linked immunosorbant assay (ELISA), chemiluminescence immunoassay and protein binding assays are used in routine testing of 25(OH)D in clinical laboratories. LCTMS (liquid chromatography tandem mass spectrometry) is the widely accepted reference method for 25(OH)D measurement. However, LCTMS is tedious, expensive and time consuming and therefore seldom used commercially.

Since vitamin D undernutrition is largely silent and subclincal, the indication for testing remains controversial. At present 25(OH)D test is the “most ordered test” in the USA. A similar trend has just begun in the upper socioeconomic stratum in India too. This clearly shows increasing awareness pertaining widespread prevalence of vitamin D deficiency among Indian clinicians. A 25(OH)D test using antibody based technologies in India costs an individual approximately INR 1500, which is unaffordable for most Indians. Surely, vitamin D status needs to be improved in most individuals in India and not just the privileged or a select few. However, testing every individual’s vitamin D levels, in a population with such a high prevalence of vitamin D deficiency is not economically and practically feasible. Furthermore, whether subclinical vitamin D deficiency in otherwise healthy individuals should be treated or not, and to what target level of serum 25(OH)D remains controversial. It will be more cost-effective to implement aggressive nationwide vitamin D supplementation and food fortification programs for the benefit of all ostensibly healthy individuals.

## 8. Vitamin D Status of Ostensibly Healthy Indians

Countrywide studies (Table 1) have reported vitamin D deficiency in as high as 70%–100% of ostensibly healthy individuals. High prevalence of vitamin D deficiency was reported from northern to southern and western to eastern India, in ostensibly healthy children, adolescents, young adults and those ≥50 years old. All over India, vitamin D deficiency was highly prevalent in pregnant women and lactating mothers. Vitamin D status of these mothers correlated well with their neonates and their exclusively breastfed infants. Subjects from rural and urban areas presented a similar picture. Relatively, fish are a rich source of vitamin D. The residents of Bengal (eastern India) eat more fish compared to the rest of the Indians. Surprisingly, their vitamin D status appears to be just as poor as in the rest of the country [54]. Similarly, even healthy young soldiers with sufficient intake of calcium, adequate sun exposure and regular exercise regimen were found to be vitamin D deficient [55,56], as were young sportswomen [57]. Among resident doctors from Mumbai (western India) [58] and also doctors from eastern India [54], most were vitamin D deficient. Vitamin D deficiency was also observed in most of 2119 healthcare professionals studied from all over India [59]. Evidently, countrywide prevalence of vitamin D deficiency is undeniable.

## 9. Correlation of Vitamin D Status with Bone Health of Ostensibly Healthy Adolescents and Adults in India

Among otherwise healthy adolescents and adults studied, along with low serum 25(OH)D levels, a significant number of subjects also revealed other biochemical and clinical manifestations of vitamin D deficiency. Biochemical evidences of suboptimal bone health are: elevated alkaline phosphatase, a surrogate marker for increased bone turnover, and elevated PTH levels (secondary hyperparathyroidism or SHPT). ALP and PTH were often reported in research papers, especially with respect to their correlation with vitamin D status. However, the most accurate and convincing evidence of bone density is DEXA. Only research articles reporting BMD, as measured by DEXA, in the context of vitamin D status of ostensibly healthy individuals are included in this section (Table 2). Vitamin D intervention studies that measured resultant BMD outcomes are discussed later in section 15 of this review.

Most studies did not show any correlation of BMD with vitamin D status. Notably, among 90 adults, who were 20–30 year-old soldiers from Indian paramilitary forces, with adequate nutrition, sun exposure and physical exercise, BMD was lower when compared to Caucasians. Among men, osteopenia was noted in 50% at the lumbar spine, 35% at the hip, and 50% at the forearm. Additionally, 10% of men had osteoporosis of the lumbar spine. Among women, osteopenia was noted in 32% at the lumbar spine, 14% at the hip and 21% at the forearm. The authors speculated that the effect of childhood malnutrition may have contributed to lower peak bone mass accumulation in these subjects [56]. BMD studies emphatically underline the need for adequate nutrition, sun exposure and physical exercise from the very beginning of one’s life, to attain peak bone mass, and later to maintain it. Indubitably, vitamin D status in India is grim and needs to be reckoned with.

## 10. Vitamin D Sufficiency via Sun Exposure Is Not a Tenable Solution for Most Indians

Vitamin D deficiency is a major health concern in India, notwithstanding the brightly shining sun. The “adequacy of exposure to sunlight of an individual’s bare skin” required to photosynthesize vitamin D is grossly ill understood. Darker skin has high melanin content which acts as a natural sunscreen. Therefore, darker skin produces a significantly lesser amount of vitamin D when compared with the individuals with fairer skin, such as Caucasians [60,61,62]. Thus, for Indian skin tone, minimum “direct sun exposure” required daily is more than 45 min to bare face, arms and legs to sun’s UV rays (wavelength 290–310 nm). With the exception of those who perforce need to work outdoors in the sun, most Indians do not get adequate sun exposure to produce sufficient amounts of vitamin D endogenously. Indian social and or religious norms related to public modesty dictate that most parts of an individual’s body, irrespective of gender, be covered. The not so D-lightful price of urbanization —in big cities a majority of people live in very high population density areas. They perforce live in overcrowded tenements, which are closely packed and 3–4 stories high. Consequently, direct sunlight does not reach inside most parts of the dwellings, thereby disallowing any sun exposure to an individual in the privacy of one’s home. Additionally, lack of space offers limited options for outdoor activities. Atmospheric pollution of metropolitan India also factors in with respect to vitamin D status [63]. Use of sunscreen creams and umbrellas do not help either. The extreme discomfort of the scorching heat associated with most sunny days of Indian summer and (not to mention) the undying desire of most Indians to attain a fairer skin complexion instantly extinguish any desire for sun exposure, and a person’s primary focus is on finding ways to avoid the sun, at all costs. In the blazing heat of India these two concerns score very high and the quest for vitamin D sufficiency takes a backseat, always. Therefore, in the Indian scenario, vitamin D sufficiency cannot be attained by depending on adequate sun exposure.

## 11. Nutritional Factors Attributing to High Prevalence of Vitamin D Deficiency in India

Vitamin D sufficiency by dietary intake is the only tenable solution for Indians. However, this solution itself has a barrage of problems.

- Most dietary sources of vitamin D have very low vitamin D content. Most of the food items rich in vitamin D are of animal origin. Most Indians are vegetarians. Commonly, a dietary source of vitamin D for vegetarians is milk, provided milk has been fortified with vitamin D. Milk is rarely fortified with vitamin D in India. The vitamin D content of unfortified milk is very low (2 IU/100 mL). Additionally, milk and milk products are unaffordable to the socioeconomically underprivileged. Another concern in India is the rampant dilution and/or adulteration of milk and milk products.
- Low calcium in Indian diet: Low dietary intake of calcium in conjunction with vitamin D insufficiency is associated with secondary hyperparathyroidism (SHPT). SHPT is further exacerbated by induced destruction of 25(OH)D and 1,25(OH)_2_D by 24 hydroxylase [64]. 24 hydroxylase is the key enzyme of vitamin D catabolism and is regulated by 1,25(OH)_2_D, PTH and FGF23 (Fibroblast Growth Factor 23) levels. FGF23 is a phosphate regulator. High serum phosphate levels increase production of FGF23 in bone osteocytes *via* the action of 1,25(OH)_2_D. Subsequently, FGF23 reduces renal phosphate resorption, indirectly suppresses intestinal phosphate absorption and also suppresses PTH and 1,25(OH)_2_D synthesis. Overproduction of FGF23 can result in increased morbidity associated with vitamin D deficiency [65]. This regulatory mechanism may explain the low 25(OH)D levels in rural subjects on a high phytate and/or low calcium diet, despite plentiful sun exposure. Most studies reported calcium intake much lower than the RDA (Recommended Daily Allowance) defined by the Indian Council of Medical Research (ICMR). Only two studies reported adequate calcium intake. In both these publications the study subjects were paramilitary soldiers [55,56]. ICMR’s RDA for calcium intake in India is lower than that of the western world.
- Calcium balance is a function of intake and excretion [66,67]. Even though the Indian diet is low in calcium content, it also has a lower protein content and therefore low endogenous acid production, which may reduce urinary calcium loss. Therefore, the amount of dietary calcium required to maintain calcium balance may be lower than for those in the Occident. The protein-induced alterations in calcium homeostasis (and possibly in bone mass) have been attributed to increments in endogenous acid production and net acid excretion due to the oxidation of the constituent sulfur containing amino acids. On the other hand, the high salt content of Indian diet is likely to increase urinary calcium excretion. A direct relation between high sodium intake and lower bone mass has been reported [68].
- Intake of caffeine from tea and coffee is very high in India. Most Indians consume milk as part of their tea or coffee. The proportion of milk is very low in these drinks. Thus calcium intake through these beverages is low. Vitamin D is stable during cooking. It is stable up to 200 °C. However, thermal stability of vitamin D is an inverse function of both temperature and time. In India, milk is boiled for several minutes before consumption. Before the same lot of milk is consumed in entirety, it is subjected to two-three rounds of boiling. In India most of the times, beverages like tea and coffee are boiled for several minutes to get the right flavor. This boiling may reduce the content of any vitamin D that there may have been left after boiling of the milk itself. Therefore, these beverages may not contribute significantly to either calcium or vitamin D intake in Indians. Vitamin D is a fairly robust vitamin. The preceding statements about its thermal degradation have been made as precautionary stance to not overstate the thermal robustness of this micronutrient. Additionally, studies have reported association of high caffeine intake with increased risk of low bone mineral density, osteoporosis, and osteoporotic fractures in middle-aged women. This situation is exacerbated in women with low calcium intake, especially in lean subjects [69].
- High prevalence of lactose intolerance in India is a major deterrent pertaining milk consumption, further lowering intake of calcium and vitamin D in these individuals. Ethnic and geographic variations of lactose intolerance were observed, with a higher prevalence in southern (Dravidian descent) and eastern India compared to northern India (Aryan descent) [70,71,72,73].
- Indian diet has high phytate content. Phytate is the principal storage form of phosphorus in many plant tissues, especially the bran portion of grains and other seeds. Phytate is indigestible to humans. Phytates chelate micronutrients such as calcium and iron, and thus reduce intestinal absorption of these nutrients. Benefits of sun exposure in rural subjects owing to an agrarian life were seen by significantly higher 25(OH)D levels [74]. However, possibly owing to high phytate content in diet, these levels were still insufficient in most individuals. Possibly, high phytate content in the diet of soldiers in northern India may have contributed to their vitamin D insufficiency, despite adequate sun exposure, nutrition and physical exercise [56].
- Notably, nearly all studies pertaining vitamin D status in healthy subjects reported a high phytate/calcium intake ratio. What Indians may require is a higher intake of calcium in their diet to lower the phytate/calcium intake ratio. Dietary habits in India have changed significantly. Many people remove a substantial proportion of bran from whole wheat flour before kneading to improve texture and fluffiness of *chapatis* (unleavened flat bread). Consumption of white bread is also very high. Most people prefer processed, split and polished pulses to whole seeds due to the ease of shorter time required for cooking and the consequent lowered expense of cooking fuel. Consumption of instant (or not) noodles and burgers also is on the rise across all socio-economic strata, with the exception of the impecunious.
- High phytate in Indian diet especially among the socio-economically lower classes stems from the elementary and immediate need of sufficiency of the calorific need. Cereals and legumes are more affordable and easily available than vegetables, milk and other dairy products. Besides, they are sources of protein for the vegetarians. Many cereals are also sources of calcium, however due to chelation by phytates its bioavailability is limited.
- In the scenario of inadequate calcium intake, vitamin D insufficiency and high phytate content in diet, environmental pollutants such as fluoride add insult to injury. Toxins like fluoride affect bone metabolism severely in the conjunction with inadequate calcium intake, especially in children [75,76].
- Cooking practices in India: Indians in general adhere to traditional cooking styles and practices, irrespective of their migration to any part of the world. In tropical climate perishable food items putrefy quickly. Notably, in India there is no perceptible government regulation on the hygiene and microbial quality control of fresh produce that reaches from the producer to the end-consumer. Consumption of uncooked fresh produce, especially vegetables, milk, *etc.*, is generally considered ill-advised. As in the rest of the world, in India too, slow cooking is widely practiced. This culinary practice, however, is ill-advised bearing in mind the thermal instability of many vitamins. As mentioned earlier vitamin D is degraded at temperatures above 200 °C. Its thermal stability is inversely related to temperature and time. Cooking gas flame reaches temperature above 1900 °C and coal stove heat reaches 300–700 °C. Water boils at 100 °C. Baking is done mostly above 175 °C but the temperature in the food does not reach such high temperatures, therefore stability of vitamin D during baking is well within acceptable range [77]. Pertaining shallow and deep-frying of food, most cooking fats and oils have smoke points above 180 °C. Shallow and deep frying of foods is very popular in India. When foods are fried, vitamin D in the food comes out into the cooking medium and is thermally degraded [78]. Pressure cooking temperatures vary depending on the pressure withstood by the cooker used and may range from 100 °C to 120 °C. Short-time (as short as possible) pressure cooking is definitely advisable to retain at least some of the thermally more stable essential nutrients in cooked food, including vitamin D.
- Protein malnutrition and poor overall nutrition resulting from poverty: Perforce, in the sordid context of poverty, focus on a balanced diet is always on the back burner. It is very convenient to attribute dietary patterns or cooking traditions, than face a reality as grim as poverty. Factually, balanced diet is only an occasional treat to the impecunious. An old adage, “When a poor man eats chicken, one of them is sick”, says it all.
- Publications indicating wide prevalence of vitamin D deficiency in healthy Indians have studied subjects mostly from lower and upper middle classes. Individuals below poverty line were not represented well in these studies. Hence, poor nutrition observed in these studies may also stem from lack of awareness of the features, benefits and necessity of balanced nutrition.

**Table 1:** Vitamin D status of ostensibly healthy Indians. All 25(OH)D values have been shown in ng/mL. To convert from nM to ng/mL, nM values were divided by (2.5). Vitamin D deficiency is defined as 25(OH)D < 20 ng/mL, insufficiency as 20–29 ng/mL and sufficiency as ≥30 ng/mL; ^‡^ Information available from the abstract of the article. Age of the subjects is in mean age (SD) years, unless otherwise indicated.

Locale	Study Subjects		25(OH)D (ng/mL) Mean (SD)	Vitamin D Status			Reference
				% deficient	% insufficient	% sufficient	
**Northern India**							
Kashmir (34.3° N)	Total *N* = 92	Adults, age 28.15 (4.9) years.	-	83	-	-	Zargar 2007 [79]
	*N* = 64	M	15.06 (12.0)	76.6	-	-	
	*N* = 28	F	5.51 (4.42)	94.4	-	-	
	*N* = 50	Urban subjects	11.26 (9.65)	85.7	-	-	
	*N* = 42	Rural subjects	12.84 (12.39)	80	-	-	
		Occupation:					
	*N* = 17	Rural/farmer, 17 M/0 F	-	70.6	-	-	
	*N* = 23	Government employee, 20 M/3 F	-	69.6	-	-	
	*N* = 15	Household, 0 M/15 F	-	100	-	-	
	*N* = 23	Medical professional, 16 M/7 F	-	91.3	-	-	
	*N* = 14	Student 11 M/3 F	-	85.3	-	-	
Punjab (31.1° N) & Haryana (29° N)	Total *N* = 90	Adults, paramilitary soldiers. Adequate diet, sun exposure and physical exercise.	-	-	-	-	Tandon 2003 [56]
	*N* = 40	M, in winter, age 22.7 (2.8) years.	18.4 (5.3)	-	-	-	
	*N* = 50	F, in summer, age 23.4 (3.1) years.	25.3 (7.4)	-	-	-	
Chandigarh (30.7° N	Total *N* = 329	Young urban adults, age 18–25 years, at the end of summer	52.9 (33.7)	-	-	72.5%	^‡^ Ramakrishnan 2011 [80]
	*N* = 237	Subjects from the same cohort at the end of winter	31.8 (21.1)	-	-	50.7%	
Delhi (28.3° N)	*N* = 31	Urban adults, M, age 25 (5) years, soldiers, winter	18.87 (4.69)	-	-	-	Goswami 2000 [55]
	*N* = 15	Urban adults, 10 M + 5 F, age 43 (16) years, depigmented persons, winter	7.28 (4.49)	-	-	-	
	*N* = 19	Urban adults, 11 M + 8 F, age 23 (5) years, physicians and nurses, winter	3.19 (1.39)	-	-	-	
	*N* = 19	Urban adults, 11 M + 8 F, age 24 (4) years, physicians and nurses, summer	7.17 (3.19)	-	-	-	
	*N* = 29	Urban adult F/mothers, age 23 (5) years, low income, summer	8.76 (4.29)	-	-	-	
	*N* = 29	Newborns of the mothers studied, 16 M + 13 F, summer	6.68 (1.99)	-	-	-	
Agota village (29° N) 80 km from Delhi	Total *N* = 57	Rural Adults	14.56 (9)	68.5	-	-	Goswami 2008 [81]
	*N* = 32	M, rural, age 42.8 (16.6) years	17.68 (9.76)	-	-	-	
	*N* = 25	F, rural, age 43.4 (12.6) years	10.76 (6.86)	-	-	-	
Delhi (28.3° N)	Total *N* = 186	Young adults F, age 18.6 (1.3) years	12.96 (9.84)	-	-	-	Marwaha 2011 [57]
	*N* = 90	Sports-girls from colleges	21.2 (7.57)	-	-	-	
	*N* = 96	College girls	5.16 (3.08)	100	0	0	
Delhi (28.3° N)	Total *N* = 642	Urban adults, middle income group	7 (4.08)	-	-	-	Goswami 2009 [82]
	*N* = 244	Adult M, age 31.4 (13.4) years	7.2 (3.64)	-	-	-	
	*N* = 398	Adult F, age 35.1 (13.4) years	6.88 (4.36)	-	-	-	
Delhi (28.3° N)	Total N = 105	Urban adults, middle income group, age 43.3 (9.7) years	9.8 (6.0)	94.3	-	-	Vupputuri 2006 [83]
	*N* = 51	M, indoor workers	10.8 (6.8)	-	-	-	
	*N* = 54	F, housewives	8.8 (4.9)	-	-	-	
Delhi (28.3° N)	Total *n* = 404	Adolescents, F, urban, age 12.3 (3.4) years	12.74 (6.17)	90.8	-	-	Puri 2008 [84]
	*n* = 193	Low income level	13.84 (6.97)	89.6	-	-	
	*n* = 211	Higher income level	11.75 (5.07)	91.9	-	-	
Delhi (28.3° N)	Total *N* = 5137	Adolescents, urban, school children, age 10–18 years	11.8 (7.2)	-	-	-	Marwaha 2005 [85]
	*N* = 3089	Lower income level, 1079 M, 2010 F	10.4 (0.4)	92.6	-	-	
	*N* = 2048	Higher income level, 968 M, 1080 F	13.7 (0.4)	84.9	-	-	
Delhi (28.3° N)	Total *N* = 664	Urban adolescent F, school girls, age 12.8 (2.7) years	11.4 (5.8)	-	-	-	Marwaha 2007 [86]
	*N* = 369	Lower income level, age 12.8 (2.7) years	11.1 (5.2)	-	-	-	
	*N* = 295	Higher income level, age 12.7 (2.6) years	11.8 (6.4)	-	-	-	
Delhi (28.3° N)	Total *N* = 1346	Urban adults ≥50 years, 643 M, 703 F, age 58 (9.5)years (range 50–84 years)	9.79 (7.61)	91.2	6.8	2	Marwaha 2011 [87]
	*N* = 995	Age group 50–65 years	9.72 (7.75)	91.3	6.9	1.8	
	*N* = 351	Age group >65 years	9.99 (7.2)	91.2	6.6	2.2	
Delhi (28.3° N)	*N* = 1346	Adults, ≥50 years, age 58 (9.5) years, 48% M, 52% F	9.8 (7.6)	91.3	6.8	1.9	Garg 2013 [88]
	*N* = 1829	Adolescents, 45% M, 55% F, age 13.3 (2.5) years	8.3 (5.2)	96.9	2.6	0.5	
Delhi (28.3°N)	*N* = 521	Pregnant women, lower-middle income level, age 24.6 (2.8) years	9.28 (4.88)	96.3	-	-	Marwaha 2011 [89]
	*N* = 342	Lactating mothers, from the above group 6–8 weeks postpartum.	7.84 (3.32)	99.7	-	-	
	*N* = 342	Exclusively breastfed Infants	8.92 (4.2)	98.8	-	-	
Delhi (28.3° N)	*N* = 180	Lactating mothers	10.88 (5.8)	-	-	-	^‡^ Seth 2009 [90]
	*N* = 180	Exclusively breastfed infants, 2–24 weeks old	11.56 (8.3)	-	-	-	
Delhi (28.3° N)	*N* = 26	Infants, urban, age 16 ± 4.1 months, 15 M/11 F, low income families, high air pollution area	12.4 (7)	-	-	-	Agarwal 2002 [63]
	*N* = 31	Infants, urban, 15.9 (3.8) months, 15 M/16 F, low income families, low air pollution area	27.1 (7)	-	-	-	
Delhi (28.3° N)		Urban slum children, age 9–30 months					Tiwari 2004 [91]
	*N* = 47	Sundernagari area, winter	38.52 (10.28)	-	-	-	
	*N* = 49	Rajiv Colony area, winter	9.5 (10.8)	-	-	-	
	*N* = 48	Rajiv Colony area, summer	7.12 (8.96)	-	-	-	
	*N* = 52	Gurgaon area, summer	7.68 (8.08)	-	-	-	
Delhi (28.3° N)	*N* = 60	Lactating mothers 25.0 (2.0) years	9.06 (4.78)	98.3	-	-	Mehrotra 2010 [92]
	*N* = 60	Breastfed infants, 3.0 (0.14) months	9.03 (4.63)	100	0	0	
Delhi (28.3° N)	*N* = 98	Lactating mothers 23.1 (3.3) years	Median 9.8 (5.0–13.8)	-	-	-	Jain 2011 [93]
	*N* = 98	Breastfed infants, 58.2% M, age 13.6 (2.2)weeks	Median 10.1 (2.5–17.1)	-	-	-	
Delhi (28.3° N)	*N* = 220	Infants, low birth-weight, at birth	Median 6.5 (4.0–54.5)	93	-	-	Agarwal 2012 [94]
	*N* = 127	Infants, low birth-weight, at 3 months	Median 11.1 (4.0–78.0)	72.4	-	-	
	*N* = 116	Infants, normal birth-weight, at birth	Median 5.8 (4.0–26.6)	94.8	-	-	
	*N* = 77	Infants, normal birth-weight, at 3 months	Median 8.2 (4–29.7)	83.1	-	-	
	*N* = 216	Mothers of low birth-weight infants, at term	Median 5.6 (4.0–38.3)	93.5	4.2	2.3	
	*N* = 116	Mothers of normal birth-weight infants at term	Median 5.8 (4.0–21.1)	96.6	1.7	1.7	
Delhi (28.3° N)	*N* = 97	Urban, lactating mothers, low income level	9.85 (6.28)	-	-	-	Agarwal 2010 [95]
	*N* = 97	Exclusively breastfed infants, age 10 weeks	12.59 ( 8.37)	-	-	-	
Lucknow, 26.8° N	*N* = 92	Urban adults, 67 F, 25 M, age 34.2 (6.7) years, hospital staff	12.3 (10.9)	78.3	-	-	Arya 2004 [96]
Lucknow (26.8° N)	Total *N* = 207	Pregnant women before labor, low and middle income group, age 24.0 (4.1) years.	14 (9.3)	-	-	-	Sachan 2005 [97]
	N = 140	Urban F	14.0 (9.5)	-	-	-	
	*N* = 67	Rural F	14.1 (8.9)	-	-	-	
	*N* = 207	Neonates/cord blood	8.4 (5.7)	95.7	-	-	
Barabanki 32 km from Lucknow (27° N)	*N* = 139	Rural pregnant women, age 26.7 (4.1) years	15.12 (7.92)	74	-	-	Sahu 2009 [98]
	*N* = 121	Rural F adolescents, age 14.3 (2.7) years.	13.32 (6.4)	88.6	-	-	
	*N* *=* 28	sisters of 34 boys, age 14.4 (2.7) years, in winter	12.52 (5.4)	-	-	-	
	*N* *=* 34	brothers of 28 girls, age 14 (3) years, in winter	27 (11.6)	-	-	-	
	*N* = 260	Rural (pregnant women + girls) in summer	22.2 (7.92)	-	-	-	
	*N* = 260	Rural (pregnant women + girls) in winter	10.92 (4.92)	-	-	-	
Varanasi (25.3° N)	*N* = 200	Adults, M, ≥50 years, age 62.61 (7.64) years	18.96 (10.23)	58	28.5	13.5	Agarwal 2013 [99]
**Southern India**							
Tirupati (13.4° N)	Total *N* = 316		-	69.3	-	-	Harinarayan 2004 [74]
	*N* = 191	Rural adults, age 44 (1.03) years	21 (0.46)	58.6	-	-	
	*N* = 125	Urban adults, age 45.5 (0.95) years	13.52 (0.59)	85.6	-	-	
Tirupati (13.4° N)	Total *N* = 1285	21% M, 79% F	-	-	-	-	Harinarayan 2008 [100]
	*N* = 205	Rural adults, age 43 years. 53% M, 47% F	M 23.73 (0.8)	M 44	M 39.5	M 16.5	
			F19 (0.89)	F 70	F 29	F 1	
	*N* = 941	Urban adults, age 46 years. 14% M, 86% F Hospital staff & relatives	M 18.54 (0.8)	M 62	M 26	M 12	
			F 15.5 (0.3)	F 75	F 19	F 6	
	*N* = 70	Rural children, age 13 years. 48% M, 52% F	M 17 (1.3)	M 76.5	M 14.7	M 8.8	
			F 19 (1.59)	F 72.2	F 13.9	F 13.9	
	*N* = 69	Urban children, age 13 years. 43% M, 57% F	M 15.57(1.2)	M 81.5	M 14.8	M 3.7	
			F 18.5 (1.66)	F 62.9	F 25.7	F 11.4	
Tirupati (13.4° N)	*N* = 164	Rural postmenopausal women, age 54 (8) years.	14.6 (7)	82	-	-	Harinarayan 2005 [101]
Tirupati (13.4° N)	*N* = 150	Semi-urban postmenopausal women, age 60.1 (5.0) years	20.85 (8.63)	50	-	-	Paul 2008 [102]
Tirupati (13.4° N)	Total *N* = 191	Semi-urban women	-	-	-	-	Harinarayan 2011 [103]
	*N* = 55	Reproductive F age 37.42 (0.72) years	15.70 (1.38)	76.3	16.4	7.3	
	*N* = 136	Postmenopausal F, age 53.29 (0.72) years.	17.70 (0.94)	66.9	22.8	10.3	
Mysore (12.3° N)	*N =* 559	Pregnant women at the 30th week of pregnancy, age 24 years.	Median 15.12 (9.6–23.4)	66.5	-	-	Farrant 2009 [104]
**Eastern India**							
Kolkata (22.5° N)	*N* = 40	Doctors, 39 M, 1 F, age 52.22 (10.91) years.	13.02 (4.77)	92.5	5	2.5	Baidya 2012 [54]
**Western India**							
Mumbai (18.9° N)	*N* = 42	Pregnant women 37th week of pregnancy, age 20–35 years, middle income group	22.99 (10.93)	-	-	-	Bhalala 2007 [105]
	*N* = 42	Cord blood/neonates	19.36 (9.57)	-	-	-	
	*N* = 35	Infants, 3 months old, exclusively breastfed	18.19 (9.74)	-	-	-	
Mumbai (18.9° N)	Total *N* = 214	Urban adults, 81% M, 19% F, age 26–30 years.	-	87.5	-	-	Multani 2010 [58]
	*N* = 174	M, Resident doctors	12.80 (7.94)	85	-	-	
	*N* = 40	F, Resident doctors	10.94 (4.54)	97.5	-	-	
Mumbai (18.9° N)	Total *N* = 1137	Young urban adults, age 30.38 (3.55) years	17.4 (9.1)	-	-	7.2	Shivane 2011 [106]
	*N* = 558	M	18.9 (8.9)	-	-	9.7	
	*N* = 579	F	15.8 (9.1)	-	-	4.8	
Pune (18.5° N)	*N* = 110	Slum toddlers, age 2.6 (0.7) years	-	-	-	-	Ekbote 2010 [107]
	*N* = 50	25 M, 25 F, outdoors (daily sun exposure of 15–60 min or more)	45.24 (31.88)	-	-	-	
	*N* = 60	31 M, 29 F, indoors	3.84 (10.64)	-	-	-	
Pune (18.5° N)	*N* = 71	Urban children, 36 M, 35 F, age 2.8 (0.6) years, all income groups	11.94 (12.61)	-	-	-	Ekbote 2011 [108]
Pune (18.5° N)	*N* = 50	Adolescent girls, low income group, age 14.7 (0.10) years	Median 9.36 (5.4–12.76)	-	-	-	Khadilkar 2010 [109]
Pune (18.5° N)		Women: housewives, working women, retired	-	-	-	-	Kadam 2010 [110]
	*N* = 80	Premenopausal F, age 45.6 (4.8) years	9.68 (4.56)	-	-	-	
	*N* = 92	Postmenopausal F, age 54.0 (7.1) years.	10.76 (6.8)	-	-	-	
Pune (18.5° N)	Total *N* = 214	Premenarchal school girls, low income group	24.6 (10.4)	34.2	-	-	Kadam 2011 [111]
	*N* = 134	Age 8–9 years	24.36 (10.32)	-	-	-	
	*N* = 80	Age 10–12 years	25.12 (10.64)	-	-	-	
**All over India**							
18 cities spread all over India	*N* = 2119	Adults, medical and paramedical personnel, 72% M, 28% F, age 42.71 (6.8) years.	14.35 (10.62)	79	15	6	Beloyartseva 2012 [59]
		No significant differences were found either between men and women, or northern and southern India.					

**Table 2:** Vitamin D status and its correlation with bone mineral density (BMD) in ostensibly healthy Indians. For additional details of vitamin D status, see Table 1. All 25(OH)D values have been shown in ng/mL. To convert from nM to ng/mL, nM values were divided by (2.5). Vitamin D deficiency is defined as 25(OH)D < 20 ng/mL. DEXA is Dual Energy X-ray Absorptiometry. Age of the subjects is in mean age (SD) years, unless otherwise indicated.

Locale	Study Subjects		BMD Measured by DEXA as Mean (SD) gm/cm^2^	Reference
**Vitamin D status correlated positively with BMD:**				
Varanasi (25.3° N)	*N* = 200	Adults, M, ≥50 years, age 62.61 (7.64) years, 58% subjects were vitamin D deficient.	At right femoral neck, 42% subjects had osteopenia and 8.5% had osteoporosis. At trochanter of right femur, 37% had osteopenia and 7.5% had osteoporosis. At right hip 41% subjects had osteopenia and 7% had osteoporosis. Mean T scores of the subjects (right femur): Neck = −0.92 (1.22), Trochanter = −0.59 (1.41) and Total hip = −0.55 (1.37). T score = (subject’s BMD-young adult mean BMD)/(1 SD of adult mean BMD) or normal BMD.	Agarwal 2013 [99]
		25(OH) D level > 22 ng/mL	normal mean BMD at femur neck, trochanter, and total hip	
		25(OH)D < 10 ng/mL	osteoporosis at femur neck and trochanter	
		25(OH) > 15 ng/mL	osteopenia at femur neck and total hip	
		25(OH) > 16 ng/mL	osteopenia at trochanter	
**Vitamin D status correlated positively with BMD, but not at all the sites studied:**				
Lucknow, 26.8° N	*N* = 92	Urban adults, M 25, F 67, 78.3% subjects were vitamin D deficient.	BMD of the spine, femur (neck, trochanter, inter-trochanter and Wards’ triangle regions) and forearm (distal, mid and ultradistal regions) was lower compared with Caucasians. 25(OH)D levels correlated with BMD at the femoral neck (*r* = 0.46, *p* = 0.037) and Ward’s triangle only. (*r* = 0.50, *p* = 0.020)	Arya 2004 [96]
Delhi (28.3° N)	Total *N* = 105	Urban adults, 51 M and 54 F, age 43.3 (9.7) years. 94.3% subjects were vitamin D deficient.	25(OH)D levels correlated with BMD at hip, but not at lumbar spine or forearm.	Vupputuri 2006 [83]
	*N* = 41	25(OH)D > 9.0 ng/mL	BMD at the hip = 0.893 (0.114) was higher than of those at 25(OH)D ≤ 9.0 ng/mL (*p* = 0.001)	
	*N* = 64	25(OH)D ≤ 9.0 ng/mL	BMD at the hip = 0.839 (0.112)	
**Vitamin D status did not correlate with BMD:**				
Punjab (31.1° N) & Haryana (29° N)	*N* = 42	Adults, age 20–30 years, 20 M, 22 F, paramilitary soldiers. Adequate diet, sun exposure and physical exercise.	Individual BMD data with peak bone mass in white Caucasians revealed that among Indian men, osteopenia was noted in 50% at the lumbar spine, 35% at the hip and 50% at the forearm. 10% of men had osteoporosis of the lumbar spine. Among women, osteopenia was noted in 32% at the lumbar spine, 14% at the hip and 21% at the forearm. In men, mean BMD was significantly lower when compared to Caucasians at the lumbar spine only. Indian men = 0.947 (0.086) *vs.* Caucasian controls = 1.091 (0.110), *p* = 0.0004.	Tandon 2003 [56]
Delhi (28.3° N)	*N* = 664	Urban adolescent F, school girls, age 12.8 (2.7) years. Lower income level *N* = 369 and Higher income level *N* = 295.	The upper income level girls were significantly taller and weighed more. They also had higher BMD in distal forearm and calcanium.BMD forearm (g/cm^2^): lower income level 0.337 (0.070) and upper income level 0.366 (0.075). BMD calcaneum (g/cm^2^): lower income level 0.407 (0.073) and upper income level 0.464 (0.093).	Marwaha 2007 [86]
Delhi (28.3° N)	*N* = 5137	Urban adolescents and school children, age 10–18 years. 92.6% children from lower income level (*N* = 3089) and 84.9% children from upper income level (*N* = 2048) were vitamin D deficient.	BMD was evaluated in 555 children. Children in the lower income level group had significantly lower BMD values at the forearm than did those in the upper income level group. Only BMD of age group (10–12) years is mentioned in this review. Boys, lower income level = 0.313 (0.044), (*N* = 27); boys, upper income level = 0.387 (0.146), (*N* = 24); girls, lower income level = 0.297 (0.048), (*N* = 70) and girls upper income level = 0.345 (0.054), (*N* = 39).	Marwaha 2005 [85]
Delhi (28.3° N)	*N* = 1600	Urban adults (M 49.5%, F 50.5%), age > 50 years. 91.2% subjects were vitamin D deficient.	35.1% subjects (M 24.6% and F 42.5%) had osteoporosis and 49.5% (M 54.3%, F 44.9%) had osteopenia.	Marwaha 2011 [112]
Delhi (28.3° N)	Total *N* = 186	Young urban college women, age 18.6 (1.3) years. 96 were college girls (control group) and 90 were sportswomen.	With the exception of femur neck, higher BMD was noted in sportswomen by 5, 13.1, 10.3, and 9.2% in total body, total hip, forearm and lumbar spine and 33% radius, respectively.	Marwaha 2011 [57]
Tirupati (13.4° N)	Total *N* = 149	Semi-urban women. 82.5% out of 40 premenopausal and 67.8% out of 109 postmenopausal women were vitamin D deficient.	Comparatively, postmenopausal women had lower BMD in forearm, hip and lumbar spine. Osteoporosis was seen at hip (15% and 28%), forearm (0% and 11%), lumbar spine antero-posterior (6% and 22%) and lumbar spine lateral (0% and 23%) among premenopausal and postmenopausal, respectively.	Harinarayan 2011 [103]
Tirupati (13.4° N)	*N* = 150	Semi-urban postmenopausal women, age 60.1 (5) years. 50% women were vitamin D deficient.	Osteoporosis was noted at lumbar spine in 48% women, at femoral neck in 16.7% women BMD: lumbar spine (LS) = 0.798 ± 0.142, femoral neck = 0.675 ± 0.108	Paul 2008 [102]
	*N* = 74	25(OH)D (>20 ng/mL)	BMD: lumbar spine = 0.816 (no SD), femoral neck = 0.694 (no SD)	
	*N* = 59	25(OH)D (10–20 ng/mL)	BMD: lumbar spine = 0.780 (no SD), femoral neck = 0.657 (no SD)	
	*N* = 14	25(OH)D (5–10 ng/mL)	BMD: lumbar spine = 0.733 (0.151), femoral neck = 0.649 (0.102)	
Mumbai (18.9° N)	*N* = 214	Resident doctors M 81.3%, F 18.7%, age 26–30 years. 87.5% subjects were vitamin D deficient.	59.7% men and 67.5% women had osteopenia. 18.39% men and 12.5% women had osteoporosis.	Multani 2010 [58]
Pune (18.5° N)	*N* = 50	Adolescent girls, low income group, age 14.7 (0.10) years.	Pune girls had lower bone area and bone mineral content, (*i.e.*, shorter and lighter girls) compared to UK South Asian and UK Caucasian girls.	Khadilkar 2010 [109]
Pune (18.5° N)	*N* = 172	Semi-urban women. Premenopausal *N* = 80, and postmenopausal *N* = 92.	Comparatively, postmenopausal women had lower BMD in femoral neck, total hip and lumbar spine. Prevalence of osteoporosis at lumbar spine was observed in 25.8% of postmenopausal women and 7.6% of pre-menopausal women. Prevalence of osteopenia was observed in 48.4% of post-menopausal and 44.3% of premenopausal women.	Kadam 2010 [110]

## 12. Genetic and Epigenetic Factors Affecting Vitamin D Status

Genetic factors leading to vitamin D deficiency in Indians, due to their effect on expression of genes that modulate vitamin D metabolism may not be ruled out. Genetic factors such as polymorphisms in 7 dehydroxylase reductase, DBP, 1 alpha hydroxylase, VDR, 25 hydroxylase, 24 hydroxylase have been studied. However, a clear association between these polymorphisms and vitamin D status is yet to be established [113,114,115]. Epigenetic factors too may be important in this context. Epigenetic factors pertain to heritable changes in the gene expression, while the DNA sequence is unchanged. These are several possibilities—post-translational modifications of histones—methylation, acetylation and phosphorylation, and also aberrant expression of microRNAs. Interaction between genetic and environmental factors, modulated by epigenetic factors has been reported. 1α hydroxylase and 24 hydroxylase have been shown to be epigenetically controlled. Information regarding association between genetic and/or epigenetic factors and vitamin D status is inconclusive and warrants further study [116].

## 13. Population Based Approaches to Improve Vitamin D Status in India: Supplementation, Food Fortification and Educational Programs

There is protracted debate ongoing on issues pertaining optimal levels of intake of vitamin D, preferred form of vitamin D for human use and extraskeletal benefits of vitamin D. However, one thing is clear—Indians need more vitamin D. Vitamin D can be obtained from three sources: sun exposure (limitations of which has been discussed earlier), vitamin D supplements and vitamin D fortified foods. There is urgent need to prioritize development of national level programs to make available, quality-regulated and affordable vitamin D supplements and vitamin D fortified foods to the Indian populace. Very importantly, the government needs to implement measures to educate the Indian populace about the current status of vitamin D in India and also the modes to attain vitamin D sufficiency.

## 14. Vitamin D Supplements Available in India

Information was collected from 10 pharmacists, from an upper middleclass locality of Delhi. Supplements commonly available are—D_3_ (cholecalciferol), 1,25(OH)_2_D_3_ and 1 alpha hydroxy vitamin D_3_ (alfacalcidol). Some formulations have calcium too. Multivitamin formulations are also available and contain about 400 IU of D_3_. None of the pharmacists had heard of D_2_ supplements. D_3_ supplement of 60,000 IU is the highest selling one and is available in powder form in sachets or as oil-based capsules. Recommended dose on the label is once per week. The sachets indicate that half a sachet per week may also be taken. According to some pharmacists, many clinicians recommended 1 sachet daily for 10 days, followed by 1 sachet/week for 5–6 weeks to 1 sachet/week forever. The other vitamin D supplements mentioned here are present in lower doses (0.25 µg or 500 IU) and daily intake (1–4 times/day) may be recommended by the clinicians. Calcium supplementation is generally recommended with vitamin D intake.

The cost of a single dose of 60,000 IU of vitamin D_3_ is about INR 30. Intake of 60,000 IU of vitamin D_3_ per week may be advisable for a short duration, for patients with severe vitamin D deficiency, but a regular weekly dose may be lead to toxicity problems. A lower dose of vitamin D not exceeding the limit of 4000 IU per day would be advisable for otherwise healthy individuals. This will also reduce the cost of the supplement and become more affordable to the common people of India.

Purchase of most drugs in India does not require a prescription. Additionally, more often than not pharmacists (commonly called “Chemists” in India) often claim to have all the information needed to prescribe medicines. Other than for acute health problems most socioeconomically backward people go directly to the pharmacist, more often than not, in order to save consultation fee of a licensed clinician. Pharmacists boldly recommended and sell medications to patients and often recommend a longer duration of medications to boost their sales. It is not uncommon for active vitamin D analogs such as calcitriol or 1-alpha D to be wrongfully prescribed by physicians and pharmacists *in lieu* of nutritional vitamin D supplements, thus putting unsuspecting patients at grave risk of hypercalcemia and if taken long-term possibly vascular calcification and kidney stones. In this context, it is very important that the along with the clinicians, pharmacists also be educated about toxicity issued related with vitamin D.

## 15. Vitamin D Supplementation Studies in Ostensibly Healthy Indians

Vitamin D supplementation studies in ostensibly healthy were compiled (Table 3). Supplementation resulted in significant improvement in vitamin D status, but a large proportion of the population had still did not attain sufficiency. The following study is noteworthy. In India, physicians often prescribe D_3_ 60,000 IU per week for 8 weeks for vitamin D deficiency. Twenty two healthy Indians with subnormal serum 25(OH)D levels were supplemented with oral D_3_ 60,000 IU/week and calcium 1 gm/day for 8 weeks. At 8 weeks the mean 25(OH)D levels increased from 10.16 (3.96) ng/mL to 22.4 (6.8) ng/mL and serum PTH normalized in all. Twenty two of the 23 subjects had 25(OH)D levels > 20 ng/mL. At the end of 12 months however, all the subjects were vitamin D deficient, once again. To sustain optimal 25(OH)D levels vitamin D supplementation would need to be ongoing after the initial loading [117].

Some supplementation studies, in ostensibly healthy adolescents and adult subjects, resulted in improved bone health. These are tabulated separately (Table 4).

More frequent and lower doses, not exceeding 4000 IU/day of vitamin D supplementation may be better for maintenance of serum 25(OH)D level. Most of the studies from India mention vitamin D status in terms serum 25(OH)D levels as below or above 20 ng/mL (or 50 nmol/L). However, one must not lose sight of the fact that the aim of all interventions in terms of vitamin D status should be ≥ 30 ng/mL or above to derive both skeletal and extraskeletal benefits of this D-lightful nutrient [118], but with a precautionary stance to not exceed 100 ng/mL.

## 16. Vitamin D Fortification in USA and Canada

Despite predominantly non-vegetarian dietary pattern, approximately 60% of the intake of vitamin D from food comes from fortified foods in USA [119] and Canada [120]. In USA, vitamin D fortification of foods is voluntary, but it is strictly regulated pertaining categories of foods, functional use and level of use, thus limiting over-fortification. Vitamin D fortified milk has been available in USA since the 1930s. Vitamin D is added to most milk sold in the United States, although it is not added to all milk products like cheese and ice cream. Some manufacturers also add it to cereal, soy milk, rice milk, and orange juice, usually along with calcium. In USA either form, D_2_ or D_3_, may be used for fortification, but commonly D_3_ is used. In Canada, the law mandates fortification of milk, milk alternatives and margarine. Similar to USA, for other permitted foods, vitamin D fortification D is voluntary, but fortification level is limited [121].

## 17. Need for Vitamin D Fortified Food Products in India

Vitamin D sufficiency via sun exposure is untenable for most Indians, as discussed earlier. Vitamin D (relatively) rich dietary sources are unaffordable and mostly limited, especially for vegetarians. Most Indians are vegetarians. Vitamin D supplements are unaffordable and not feasible as a population based approach. Fortification of widely consumed staple foods with vitamin D is the only viable solution towards attaining vitamin D deficiency in India. Unlike supplementation strategies, fortification of food with vitamin D poses a negligible risk of toxicity.

## 18. Feasibility of Fortification of Foods with Vitamin D in India

Food fortification is a much more economically viable approach compared to vitamin D supplementation. While the cost of fortified food items will be more than unfortified foods, it will be lower than supplementation.

1. Adaptability to fortified food by the consumers is much better than to supplementation. Food fortification requires relatively less change in food habits and preferences, leading to better efficacy of fortification programs, lowered cost to the consumer and a larger profit to the food manufacturers. Clearly a win-win partnership for all.
2. Food fortification may be a better choice compared to supplementation strategies, especially when targeting those who need it the most—women (including non-pregnant, pregnant and lactating), infants, children (especially girls, who are sidelined, more often than not, in India) and senior citizens.
3. Use of staple foods such as *chapati* flour, rice, *etc.*, for fortification may have certain advantages over other fortification matrices. Indian government offers cereals, *chapati* flour, rice, lentils, *etc.*, at subsidized rates to the socioeconomically underprivileged citizens. Additionally, cost of these products is generally tightly regulated by the government. Thus, no large fluctuations are observed in the cost of these food items in the open market also. This will ensure continued consumption of these foods fortified with vitamin D, by those who avail of them and result in better and sustained economical feasibility of the fortification programs in the long run.
4. Two vitamin D fortification studies in ostensibly healthy subjects were reported in the literature (see Table 5). Underprivileged toddlers, fed with fortified *laddoos*, resulted in significant increase in serum calcium and vitamin D levels and also in total body less head (TBLH) bone mineral content (BMC) [122]. The cost of fortified *laddoo* was INR 20 per *laddoo*, which may be prohibitive in itself. More cost effective food items could be fortified with vitamin D. In another study, 776 subjects (boys and girls) were given fortified milk, which resulted in significant improvement in their vitamin D status [123]. These results support the strategy of fortification of foods in India for redressing malnutrition problems in India.

**Table 3:** Vitamin D supplementation studies in ostensibly healthy Indians. All 25(OH)D values have been shown in ng/mL. To convert from nM to ng/mL, nM values were divided by (2.5). Vitamin D deficiency is defined as 25(OH)D < 20 ng/mL, insufficiency as 20–29 ng/mL and sufficiency as ≥30 ng/mL. ^‡^ Information available from the abstract of the article. D_3_ is cholecalciferol. Calcium amount mentioned is of elemental calcium. Age of the subjects is in mean age (SD) years, unless otherwise indicated.

Locale	Study Subjects and Supplementation Protocols	Vitamin D Status										Reference
		Before		After								
		25(OH)D Mean (SD)	25(OH)D Mean (SD)	25(OH)D Mean (SD)				% Deficient				
Barabanki, 32 km from Lucknow (27° N)	Total *N* = 84, Rural pregnant women, low income group, all received 1 gm calcium/day	Median 12.92 (9.12–20.04)	-	-				-				Sahu 2009 [124]
	*N* = 14, Control group	Median 10.32 (7.56–12.28)	-	Median 9.52 (6.88–13.04)				-				
	*N* = 35, Group A, 60,000 IU D_3_ in the 5th gestational month	Median 13.26 (9.04–19.08)	-	Median 12.36 (9.92–19.24)				-				
	*N* = 35, Group B, 120,000 IU D_3_ in the 5th and 7th gestational month	Median 16.04 (10.76–23.36)	-	Median 21.36 (16.46–35.02)				-				
Lucknow (26.8° N)	Pregnant women and neonates. Women age 26.7 (4.0) years, low and middle income group. All were prescribed 1 gm calcium/day	-	-	-				-				Kalra 2012 [125]
	*N* = 48, Control group	-		Median 15.68 (8.48–29.36)				-				
	*N* = 33, Neonates of control group	-	-	Median 7.4 (4.04–11.88)				-				
	*N* = 48, Group A, 60,000 IU D_3_ in 2nd trimester	Median 12.68 (5.8–18.28)	-	Median 10.48 (7.08–23.08)				-				
	*N* = 31, Neonates of group A	-	-	Median 11.28 (6.04–17.2)				-				
	*N* = 48, Group B, 120,000 IU D_3_ in 2nd and 3rd trimester	Median 12.8 (5.8–18.28)	-	Median 23.48 (15.36–35.76)				38				
	*N* = 28, Neonates of group B	-	-	Median 9.64 (4.88–17.56)				-				
Delhi (28.3° N)	Low birth weight term infants, from all income groups.	-	-	-				-				Kumar 2011 [126]
	*N* = 237, Control group	-	-	14.4 (10.2)				73.4				
	*N* = 216, Study group, started at 7 days after birth, 1400 IU D_3_ (dissolved in breast milk) per week, given for 6 months	-	-	22.0 (9.0)				43.5				
Delhi (28.3° N)	Low birth weight term infants, from all income groups.	-	-	-				-				Trilok-Kumar 2012 [127]
	*N* = 187, Control group	-	-	15.1 (10.5)				72.19				
	*N* = 164, Study group, started at 7 days after birth, 1400 IU D_3_ (dissolved in breast milk) per week, given for 6 months	-	-	22.8 (8.9)				37.8				
Delhi (28.3° N)	Urban adolescent schoolgirls	-	93.7	-				-				Marwaha 2010 [128]
	*N* = 60, low income group, age 12 (2.8) years, 60,000 IU D_3_/2 months, for 1 year	12.48 (0.67)	98	21.2 (1.22)				38				
	*N* = 64, low income group, age 11.4 (3) years, 60,000 IU D_3_/month, for 1 year	13.17 (0.54)	97	23.73 (1.05)				28				
	*N* = 81, high income group, age 11.6 (2.7) years, 60,000 IU D_3_/2 months, for 1 year	11.65 (0.61)	94	15.3 (0.85)				80				
	*N* = 85, high income group, age 11.7 (2.8) years, 60,000 IU D_3_/month, for 1 year	12.32 (0.55)	88	19.97 (0.80)				57				
Delhi (28.3° N)	Total *N* = 482, Adolescents, after 60,000 IU D_3_/week, as per each group. All subjects in all groups received 600 IU D_3_/day for 12 weeks	-	-	-				-				^‡^ Garg 2013 [129]		
	Group A, 60,000IU D_3_/week for 4 weeks	-	-	-				-						
	Group B, 60,000IU D_3_/week for 6 weeks	-	-	27.0 (6.6)				-						
	Group C, 60,000IU D_3_/week for 8 weeks	-	-	28.0 (8.7)				-						
Delhi (28.3° N)	*N* = 172, Urban young women, nursing students, age 21.7 (4.4) years	9.3 (3.37)	-	-				-				Goswami 2012 [130]		
	*N* = 43, Group A, double placebo	8.6 (3.26)	-	7.7 (3.64)				-						
	*N* = 43, Group B, 1 gm calcium/day	9.9 (3.24)	-	8.1 (2.92)				-						
	*N* = 43, Group C, 60,000 IU D_3_/week for 8 weeks, followed by 60,000 IU D_3_ twice/month for 4 months	9.2 (3.40)	-	29.9 (8.35)				-						
	*N* = 43, Group D, 1 gm calcium/day. Also 60,000 IU D_3_/week for 8 weeks, followed by 60,000 IU D_3_ twice/month for 4 months	9.5 (3.47)	-	27.0 (9.54)				-						
Delhi (28.3° N)	Urban adults, medical students, age 31.5 (5) years	-	100	-				-				Gupta 2010 [131]	
	*N* = 20, Control group, 11 M, 9 F, double placebo	8.44 (3.76)	-	11.88 (6.0)				-					
	*N* = 20, Study group, 13 M, 7 F, received 1 gm calcium/day for months and 60,000 IU D_3_/week for 8 weeks and then once per month for 4 months	10.16 (3.96)	-	22.4 (6.8)				-					
Delhi (28.3° N)	*N* = 23, Urban adults, age F 33.9 (13.1) years, age M 34 (17) years. 60,000 IU D_3_/week for 8 weeks. 1 gm calcium was given daily for 8 weeks. Subjects measured at 8 weeks.	5.4 (1.2)	100	32.96 (8.29)				4.3				Goswami 2008 [117]	
	The subjects were measured again at 12 months.			9.88 (4.6)				100					
Delhi (28.3° N)	Urban postmenopausal women, age 54.8 (6.7) years. All subjects in all groups received calcium 1 gm/day for 3 months.	-	83.7%	-				-				Agarwal 2013 [132]	
	*N* = 21, Control group A	12.99 (6.74)	-	8.0 (5.28)				95.3					
	*N* = 25, Group B, 500 IU/day D_3_ for 3 months	12.92 (8.20)	-	13.34 (9.52)				84.0					
	*N* = 18, Group C, 1000 IU/day D_3_ for 3 months	14.38 (11.07)	-	23.71 (11.71)				33.33					

**Table 4:** Vitamin D supplementation studies reporting improved bone health post-intervention, in ostensibly healthy Indians (adolescents and adults). All 25(OH)D values have been shown in ng/mL. To convert from nM to ng/mL, nM values were divided by (2.5). Vitamin D deficiency is defined as 25(OH)D < 20 ng/mL, insufficiency as 20–29 ng/mL and sufficiency as ≥30 ng/mL. D_3_ is cholecalciferol and D_2_ is ergocalciferol. Calcium amount mentioned is of elemental calcium. Age of the subjects is in mean age (SD) years, unless otherwise indicated.

Locale	Study Subjects and Supplementation Protocols	Vitamin D Status			Reference
		Before	After		
		25(OH)D Mean (SD)	25(OH)D Mean (SD)	Bone Health Status	
Pune (18.5°N)	Urban, postmenarchal schoolgirls, age 14–15 years, low income group. All the girls were shorter and lighter compared to Indian norms.	-	-	-	Khadilkar 2010 [133]
	*N* = 24, Control group: placebo and 250 mg calcium/day for 1 year.	Median 8.32 (5.08–12.16)	Median 11.24 (6.68–13.6)	Mean increase in 25(OH)D was 19%, mean decrease in PTH was 9%. BMD showed no change.	
	*N* = 25, Study group: 300,000 IU D_2_ once every 3 months and 250 mg calcium/day for 1 year. Study group was divided into sub groups: (1) girls who had menarche within 2 years and (2) girls who had menarche for over 2 years, at the start of the study.	Median 9.8 (5.08–13.28)	Median 30.08 (25.68–34.2)	Overall, 25(OH)D increased by 68% and PTH decreased by 27%. No significant changes were observed in subgroup (2). However, in subgroup (1) total bone mineral content (TB BMC) and total bone area (TB BA) increased and lumbar spine BMC was higher.	
Pune (18.5°N)	Urban, premenarchal schoolgirls, age 9.8 (1) years. All the girls were shorter and lighter compared to Indian norms. All subjects received 300,000 IU D_3_ once every 3 months, for 1 year.	65.7% vitamin D deficient	-	TB BMC, TB BA, TB BMD, TB LBM (lean body mass) and TB fat% increased significantly in all three groups over baseline. There was no significant difference between the results of study groups (Ca) and (Ca + MZ).	Khadilkar 2012 [134]
	*N* = 69, Control group, two placebos.	23.08 (10.68)	-	Total body BMC increased by 17.6%,	
	*N* = 70, Study group (Ca), 500 mg calcium 6 days/week, for 1 year.	24.76 (10.36)	-	Total body BMC increased by 22.3%.	
	*N* = 71, Study group (Ca + MZ), 500 mg calcium and multivitamin + zinc (Becosule-Z^®^) 1 tablet 6 days/week, for 1 year.	26.36 (10.92)	-	Total body BMC increased by 20.8%.	

**Table 5:** Vitamin D food fortification studies in ostensibly healthy Indians. All 25(OH)D values have been shown in ng/mL. To convert from nM to ng/mL, nM values were divided by (2.5). Vitamin D deficiency is defined as 25(OH)D < 20 ng/mL, insufficiency as 20–29 ng/mL and sufficiency as ≥30 ng/mL. Age of the subjects is in mean age (SD) years.

Locale	Study Subjects and Fortification Protocols	Vitamin D Status							Reference
		Before				After			
		25(OH)D Mean (SD)	% Deficient		% Sufficient	25(OH)D Mean (SD)	% Deficient	% Sufficient	
Delhi (28.3° N) Northern India	Total *N* = 713, Urban school children and adolescents, age 11.74 (1.05) years, 300 M, 413 F. All were given unfortified milk for 12 weeks and then divided into 3 groups.	11.69 (5.36)	92.3		0.7	20.44 (9.88)	-	-	Khadgawat 2013 [123]
	*N* = 237, Control group: Unfortified milk 200 mL/day for 12 weeks, 102 M, 135 F	11.74 (5.23)	93.6		-	10.83 (5.24)	94.0		
	*N* = 243, Study Group A, fortified milk, 600 IU D_3_/200 mL/day for 12 weeks, 97 M, 146 F	11.42 (5.24)	95.0		1.23	22.87 (6.75)	30.0	12.34	
	*N* = 233, Study Group B, fortified milk, 1000IU D_3_/200 mL/day for 12 weeks, 101 M, 132 F	11.94 (5.62)	87.9		0.42	27.67 (8.47)	18.9	36.05	
Pune (18.5° N) Western India	Total *N* = 58, Urban children, low income group, age 2.7 (0.52) years. While both groups received D_3_ fortified laddoos. Control group received only 156 mg of calcium, as opposed to 405 mg of calcium to the study group.								Ekbote 2011 [122]
	*N* = 28, Control Group, 1 fortified *laddoo* *, 156 mg of calcium, 5 times/week for a year. Also given, 1 laddoo with 30,000 IU D_3_/month for 1 year.	7.56 (10.8)	84	-		23.24(4.76)	-	-	
	*N* = 30, Study Group, 1 fortified laddoo, 405 mg of calcium, 5 times/week for a year. Also given, 1 laddoo with 30,000 IU D_3_/month for 1 year.	10.0 (10.84)	83	-		25.75 (9.56)	-	-	

* A *laddoo* is an Indian cereal-legume snack, spherical, about 4 cm in diameter and weight 50 g. The ingredients for unfortified laddoos, were ragi (*Eleusine coracana*) and whole dried Bengal gram (*Cicer arietinum*) 7.5 g each, sesame seeds (*Sesamum indicum*) and poppy seeds (*Papaner somniferum*) 5 g each, 5 g of clarified butter (Ghee) and 15 gm of refined palm sugar.

## 19. Food Items Which Could Be Fortified with Vitamin D in India

1. Milk: The whole array of different grades of milk available could be fortified—whole milk, toned, double toned and skim milk.
2. Milk curd and yogurt.
3. Infant formulas.
4. Butter, *ghee* (clarified butter) and oils, to use as spreads or to spike already cooked food.
5. Soy milk, soy curd (tofu), orange juice and mango juice may be fortified to cater to the needs of the lactose intolerant individuals and those who are allergic to milk proteins. Processed cheese also has very low lactose content and is rich in calcium and may be fortified for the benefit of the lactose intolerant. Due to high prevalence of dyslipidemia, metabolic syndrome and cardiovascular diseases in India, these fortified items will also offer healthier choices to the general population.
6. Widely consumed and affordable staple food items such as *chapati* flour, *maida* (all purpose wheat flour, used to make bread and other bakery products), rice and rice flour may be suitable vehicles for fortification strategies in the Indian scenario.

## 20. Foods Fortified with Vitamin D Available in the Indian Market

Vitamin D fortified milk from Amul^®^ (an Indian dairy cooperative, located in Anand, Gujarat, India) is the only fortified milk product found in the general market. It is 4.5% fat, homogenized milk fortified with calcium 150 mg, vitamin A 75 μg and vitamin D 0.5 μg (20 IU), *etc*., per 100 mL. The expiry date of this milk is 120 days if the carton is unopened. Incidentally, with a 10% or more loss per month at 4 °C, there is not much vitamin D left by 120 days. It may be hoped that storage temperatures are always adhered to. But in India this is a remote possibility due to economical and technical limitations. In a brief survey, most retailers reported that the Amul^®^ milk cartons supplied to them were generally one month past expiry date already at the time of delivery and that the demand for this product was very low. Cost per liter is INR 48 (as on 12 January 2013) as opposed to the cost of unfortified milk (INR 30).

Kellogg’s breakfast cereals fortified with vitamin D along with other micronutrients are also available. However, the exorbitant prices of these products are essentially prohibitive for consumption by the common people of India.

## 21. Estimated Burden of Bone Diseases on Indian Healthcare System

There is no “national body” instituted by the government of India, to study and document the prevalence of bone diseases in India. The following figures pertaining bone health were perforce taken from research articles. In India, bone diseases such as rickets, osteomalacia and osteoporosis have been [67,135,136,137] and still are widely prevalent and formidable problems of our nation. As per 2001 Census Report about 163 million people were above the age of 50 years. This figure will be much bigger from the 2011 Census Report and may exceed 200 million. About 15%–20% of persons above the age of 50 years would be developing osteoporosis [136]. As presented in this review, BMD studies of ostensibly healthy Indians show that a significant proportion of younger Indians too are suffering from this silent disease. As the early signs of osteoporosis are diagnosed with accuracy, BMD data will add to these already astounding figures. Some staggering figures and estimates pertaining rickets, osteomalacia and osteoporosis are mentioned in some reviews [136,137].

Vitamin D status in India is grim not only in the lower and but also in the upper socioeconomic classes. For the sake of simplicity and argument, let us consider only the skeletal benefits of vitamin D. Vitamin D sufficiency in women of reproductive age would at the very least result in —improved women’s health, improved outcome of childbirth and birth of infants who may have a better chance of a healthy existence from the beginning of their lives. Vitamin D sufficiency in growing children would mean allowing for their full growth potential—physically, mentally and psychologically. In children, rickets translates into life-long deformity, osteomalacia—a painful and subpar existence. Osteoporosis is a silent disease tethered to a constant threat of fractures to the unbeknownst afflicted. In elderly patients, especially hip fractures result in the end of an independent existence. Notably, most people in India do not have health insurance. Hospitalization of patients with hip fractures is much longer than for those being treated for cancers. For those who cannot afford hospitalization for treatment, it is a long, agonizing and often an unending wait for resurrection. This fact indicates the heavy load on the healthcare system and the economy of the nation due to bone diseases alone.

## 22. Government’s Intervention Required

Vitamin D sufficiency status may not be treated as a “feel good status” for the affluent who can afford medical expenses and expensive vitamin D supplements. Vitamin D is prevalent across all socioeconomic strata. It is imperative that policymakers understand the gravity of the situation pertaining vitamin D status and as a consequence—the untold burden on the healthcare system in India. The information presented in this article ought to persuade policymakers to take substantive measures towards attaining vitamin D sufficiency. Actions needed to be taken by the government of India are as follows:

1. Revision of RDA (Recommended Daily Allowance) values for vitamin D and calcium intake, by ICMR (Indian Council of Medical Research) is imperative.
2. Educational Programs: Vitamin D deficiency is the world’s most under-diagnosed and under-treated disease. There is great need for awareness of its implications among physicians and the general public at large. Adequate investment of money, time and effort is required to develop, launch and sustain public awareness programs.
  2.1 The curricula of medical colleges need to be updated pertaining vitamin D status of the Indian populace. Inclusion of pertinent and updated information pertaining vitamin D, and its skeletal and extraskeletal benefits is required.
  2.2 Making bone and mineral health a priority: All healthcare facilities, including all primary health care facilities should institute awareness programs to educate the physicians and the local residents about the need for vitamin D sufficiency.
  2.3 Active participation of social organizations is required. Social workers and school teachers need to be duly informed and educated, so they can spread the word about necessity for vitamin D sufficiency and the ways to achieve it.
  2.4 Aggressive government sponsored mass media programs, with the aid of tele-media and print media are required to educate the masses about the grim vitamin D deficiency status in India. The masses should be educated on the benefits of a combination of sun exposure, vitamin D fortified food items, supplements and regular physical exercise.
  2.5 A need for sufficient calcium intake along with vitamin D must be stressed at all times.
3. Vitamin D supplements: Affordable, good quality and readily available vitamin D supplements for the masses are needed. Vitamin D supplements should be made available at all primary care health centers to pregnant women and lactating women.
4. Fortification of foods with vitamin D: First and foremost possibility thinking is required. Indian government has successfully launched food-fortification programs before. The most visible result to the public eye being—iodized salt. Fortification of food with vitamin D is doable. Political and administration’s will and support should be available from the development stage of the fortification program and equally importantly sustained.
  4.1 Involvement of the food industry by encouraging private enterprises operating at the national and local level is required. Support in terms of technical expertise pertaining production of fortified food items, availability of standardized vitamin D formulation(s) and information on the marketing potential of the fortified items should be made available.
  4.2 Effective legislation to ensure good quality and regulated vitamin D fortified foods at minimal cost to the end consumer is required. That said, both the administration and legislature should very importantly be facilitative and not be restrictive or punitive to those who are striving towards attaining better bone and mineral health of the masses.
5. School going children could benefit from the following:
  5.1 Educators should emphatically teach the need for vitamin D sufficiency and benefits of a healthy lifestyle. Inclusion of the benefits of vitamin D in the ken of the kids may perchance spread this information to their (unaware) kin too.
  5.2 Mandatory distribution of vitamin D fortified foods at midday meals in schools.
  5.3 Daily physical exercise should be made compulsory in schools.
6. Affordable and widely accessible testing facilities for vitamin D levels should be made available to individuals who are at high risk of clinical vitamin D deficiency.
7. Reliable and sensitive technology such as DEXA should be made available at minimal cost for screening bone mineral density in individuals at high risk, with easy accessibility, throughout India.
8. Last, but not the least, government should extend support to research groups, so that the impact of supplementation programs and fortification strategies in actual practice may be studied and monitored and more efficacious ways developed. Continued research and epidemiological studies are required, to clearly state the enormity of the vitamin D deficiency status and its implications on general health of Indians, countrywide. Vitamin D status from western, eastern, northeastern India and the Himalayan regions and are still either few or lacking. Also, the very poor and the marginalized citizens have not been included in such studies.

## 23. A Lagniappe for the Readers: Other Countries of the Indian Subcontinent Present a Similar Scenario Pertaining Vitamin D Status

Other countries of the Indian subcontinent: Pakistan, Bangladesh, Nepal, Sri Lanka, Myanmar and Bhutan share the same geographic locale and socioeconomic culture with India. Data were compiled from these countries too. Vitamin D deficiency is highly prevalent in these countries (Table 6). One report was available from Pakistan pertaining bone health of ostensibly healthy subjects. In a cohort of 140 rural postmenopausal women from Khazana (34.7° N) and Nahaqi (34.2° N), age 52(SD 2) years, 42% had osteopenia and 28% had osteoporosis. 25(OH)D levels correlated with BMD [138]. No reports were available from other countries of the Indian subcontinent pertaining vitamin D status and its correlation with BMD. One supplementation study from Bangladesh showed significant improvement in the vitamin D status (Table 7). One supplementation study from Pakistan showed significant improvement in the vitamin D status and improved bone health (Table 8). No food fortification studies were available from any of these countries. From the available data, it is clear that vitamin D scenario in these countries is similar to that in India. The data from other countries of the Indian subcontinent also show that though India may be economically more advanced that some of its neighbors, the vitamin D scenario is similar all over the subcontinent.

## 24. Conclusions

Widespread prevalence of vitamin D deficiency in India is undeniable. Factually, sun exposure is an untenable solution, for most individuals in India, towards attaining vitamin D sufficiency. Low calcium intake in conjunction with vitamin D deficiency makes matters worse. The need for improvement in vitamin status of the Indian population is both important and urgent. The Indian government needs to take substantive measures in this direction. Revision of RDA for calcium and vitamin D is required. Better facilities and technologies should be made available countrywide to enable timely diagnosis of clinical manifestations of vitamin D deficiency in individuals who need attention by the clinicians. Population-based programs at the national level must be developed to increase awareness of the problem at hand, to provide affordable vitamin D supplements and also to provide vitamin D fortified foods to the Indian populace at large. Research in this field needs continued support to provide a comprehensive picture of the ongoing vitamin D problem and also to study and monitor the effect(s) of a partnership between the government, healthcare system, industry and consumers, aimed at improving the vitamin D status in India.

**Table 6:** Vitamin D status of ostensibly healthy subjects from other countries of the Indian subcontinent. All 25(OH)D values have been shown in ng/mL. To convert from nM to ng/mL, nM values were divided by (2.5). Vitamin D deficiency is defined as 25(OH)D < 20 ng/mL, insufficiency as 20–29 ng/mL and sufficiency as ≥30 ng/mL. Age of the subjects is in mean age(SD) years, unless otherwise indicated.

Locale	Study Subjects		25(OH)D (ng/mL)Mean (SD)	Vitamin D Status			Reference
				% Deficient	% Insufficient	% Sufficient	
**PAKISTAN**							
Lahore (31.5° N) & Rawalpindi (33.6° N)	*N* = 92	Adults, median age 29 (11–75) years, rural & urban.	Median 29.7 (3.0–63.1)	-	-	-	Rashid 1983 [139]
	*N* = 48	M	33.0 (11.0)	-	-	-	
	*N* = 44	F	27.0 (11.3)	-	-	-	
Karachi (24.8° N)	Total *N* = 123	Urban adults, age 32.7 (8.7) years, hospital staff	16.44 (3.84)	69.9	21.1	8.9	Mansoor 2010 [140]
	*N* = 53	F	14.88 (9.2)	-	-	-	
	*N* = 70	M	17.64 (3.88)	-	-	-	
Karachi (24.8° N)	*N* = 305	Urban adults, F, low, middle and high income groups equally represented, age 31.97 (8.00) years	8.70 (8.64)	90.5	5.2	4.3	Khan 2012 [141]
Karachi (24.8° N)	*N* = 50	Pregnant women, age 28.16 (4.4) years, low and middle income group	24.1 (11.70)	46	32	22	Karim 2011 [142]
	*N* = 50	Cord blood/neonates	20.4 (10.99)	-	-	-	
Karachi (24.8° N)	*N* = 75	Pregnant women at term, age 26 (6.5) years, low income group	Preterm 16.89 (7.83)Term 13.19 (6.74)	72	17	11	Hossain 2011 [143]
	*N* = 75	Cord blood/neonates	Preterm 21.91 (9.73)Term 15.81 (9.56)	72	17	11	
Karachi (24.8° N)	*N* = 62	Exclusively breastfed Infants	13.83 (10.62)	-	-	-	Atiq 1998 [144]
	*N* = 62	Mothers	12.8 (8.98)	-	-	-	
	*N* = 37	Breastfed infants, age 6 weeks–11 months	8.98 (6.56)	-	-	-	
	High income	Mothers, housewives, mostly indoors	10.6 (9.64)	-	-	-	
	*N* = 25	Breastfed infants, age 6 weeks–11 months	20.93 (15.7)	-	-	-	
	Low income	Mothers, working	15.90 (6.03)	-	-	-	
**BANGLADESH**							
Dhaka (23.7° N)	*N* = 99	Adults, F, low income level, age 16–40 years, 88% housewives	Median 14.68(range 10–48)	-	-	-	Islam 2002 [145]
	*N* = 90	Adults, F, high income level, age 16–40 years, 76% housewives	Median 17.4(range 10–48)	-	-	-	
Dhaka (23.7° N)	*N* = 36	Adults, F, high income level, age 22.3 (1.9) years, unveiled	12.12 (9.04)	-	-	-	Islam 2006 [146]
	*N* = 30	Adults, F, medium income level, age 47.7 ( 9.4) years, veiled/*purdah*	12.4 (4.4)	-	-	-	
Dhaka (23.7° N)	*N* = 200	Adults, F, urban factory workers, low income group, age 22.6 (3.7) years	14.68 (4.48)	88.5	-	-	Islam 2008 [147]
Sylhet (24.9° N)	*N* = 29	Infants, age 71 (32) days, 25 M, 4 F, rural, low income group	14.68 (6.84)	-	-	-	Roth 2010 [148]
Matlab (23.3° N)	*N* = 98	Cord blood, neonates of rural women/mothers, age 25.8 (5.9) years	Median 23·8 (18·6–30·8)	-	-	-	Doi 2011 [149]
Dhaka (23.7°N)	*N* = 76	Pregnant women, 20 weeks of gestation, age 21.55 (4) years	24.86 (1.02)	-	-	34.21	Ullah 2013 [150]
**NEPAL**							
Sarlahi (26.5° N)	*N* = 1163	Rural F, pregnant, age 23.6 ± 6.0 years, gestational age 10.9 (4.6) week.	20.44 (9.85)	-	-	-	Jiang 2005 [151]
**SRI LANKA**							
Kandy (7.2° N)	*N* = 85	Adults, M, age 47.0 (8.3) years.	25.2 (9.12)	34·1	-	-	Meyer 2008 [152]
	*N* = 111	Adults, F, age 46.4 (7.8) years.	18.96 (6.32)	58·6	-	-	
Galle (6.2° N)	*N* = 122	Preschool children, M, age 45.93 (9.8) months	34.8 (13.4)	-	-	-	Hettiarachchi 2012 [153]
	*N* = 126	Preschool children, F, age 46.56 (8.5) months	32.62 (14.8)	-	-	-	
No pertinent reports were available from Myanmar and Bhutan.							

**Table 7:** Vitamin D supplementation studies in ostensibly healthy subjects from other countries of the Indian subcontinent. All 25(OH)D values have been shown in ng/mL. To convert from nM to ng/mL, nM values were divided by (2.5). Vitamin D deficiency is defined as 25(OH)D < 20 ng/mL, insufficiency as 20–29 ng/mL and sufficiency as ≥30 ng/mL. D3 is cholecalciferol.

Locale	Study Subjects and Supplementation Protocols	Vitamin D Status				Reference
		Before		After		
		25(OH)D Mean (SD)	% Deficient	25(OH) DMean (SD)	% Deficient	
**PAKISTAN** One report is included in Table 8						
**BANGLADESH**						
Dhaka (23.7° N)	Pregnant women, age 22.4 (SD 3.5) years, 26–29 weeks of gestation,	-	-	-	-	Roth 2013 [154]
	*N* = 80, Control group, placebo	17.6 (8.36)	66.25	15.36 (7.24)	79.3	
	*N* = 67, Cord blood/neonates of control group	-	-	15.6 (7.48)	80.5	
	*N* = 80, Study group, received 35,000 IU D3/week until delivery	18.16 (7.36)	62.5	53.76 (12.28)	0	
	*N* = 65, Cord blood/neonates of study group	-	-	41.12 (11.44)	4.6	
No pertinent reports with information required were found from Nepal, Sri Lanka, Myanmar and Bhutan.						

**Table 8:** Vitamin D supplementation studies reporting improved bone health post-intervention, in ostensibly healthy subjects from other countries of the Indian subcontinent: All 25(OH)D values have been shown in ng/mL. To convert from nM to ng/mL, nM values were divided by (2.5). Vitamin D deficiency is defined as 25(OH)D < 20 ng/mL, insufficiency as 20–29 ng/mL and sufficiency as ≥30 ng/mL. D3 is cholecalciferol. Calcium amount mentioned is that of elemental calcium.

Locale	Study Subjects and Supplementation Protocols	Vitamin D Status			Reference
		Before	After		
		25(OH)D Mean (SD)	25(OH)D Mean (SD)	Bone Health Status	
**PAKISTAN**					
Lahore (31.5° N)	*N* = 53, premenopausal women, age 41.3 (SD 8.2) years. All subjects had anterior tibial tenderness (ATT). Subjects received a total of 1,800,000 IU intramuscular D3, in 3 doses of 600,000 IU each, on alternate days. All subjects received 1.2 gm calcium/day for 3 months.	12.1 (17.5) 81.1% were vitamin D deficient	51.9 (no SD)	Significant improvement in ATT was observed. 88.7% women attained vitamin D sufficiency and 92.4% had normalization of PTH levels. Change in ATT correlated with change in PTH, but did not correlate with 25(OH)D levels.	Ali 2013 [155]
No pertinent reports with information required were available from Bangladesh, Nepal, Sri Lanka, Myanmar and Bhutan.					
